# Weight-Based Victimization and Physical Activity Among Adolescents With Overweight or Obesity: A Scoping Review of Quantitative and Qualitative Evidence

**DOI:** 10.3389/fspor.2022.732737

**Published:** 2022-01-28

**Authors:** Ingeborg B. Skogen, Finn Ove Båtevik, Rune Johan Krumsvik, Kjetil L. Høydal

**Affiliations:** ^1^Department of Physical Education, Faculty of Arts and Physical Education, Volda University College, Volda, Norway; ^2^Department of Social Work, Volda University College, Volda, Norway; ^3^Department of Education, Faculty of Psychology, University of Bergen, Bergen, Norway

**Keywords:** weight-based victimization, physical activity, overweight, obese, adolescents, weight stigma

## Abstract

**Background:**

Increased physical activity engagement plays a vital role in preventing and treating overweight or obesity in children and adolescents. Research has found that adolescents who are overweight or obese tend to participate in less physical activity than adolescents of average weight. Weight-based victimization (victimization related to weight or body size) is highly prevalent in adolescence and seems to be a factor that might negatively impact engagement in physical activity. This scoping review's objective was to identify the nature and extent of research evidence on links between weight-based victimization and physical activity among community-based or primary health care samples of adolescents who are overweight or obese.

**Methods:**

Following established scoping review methods, we searched electronic databases PubMed, Web of science, SportDiscuss, and Cinahl from 23 Nov 2020 to 02 Dec 2020. Published studies with community-based or primary health care samples of adolescents who were overweight or obese (aged 13–18 years, Iso-BMI ≥ 25, i.e., age and gender specific percentiles based on specified cut-off value for overweight and obesity) and that were peer-reviewed and published were included in the analysis. Data from the included studies were put into a table and analyzed descriptively and numerically in terms of the extent and nature of the studies.

**Results:**

After full text review, 12 articles were included, equally distributed in the qualitative and quantitative paradigm. There was a homogeneity of the research designs applied. Articles revealed high frequency of weight-based victimization. Among quantitative studies the reported links between weight-based victimization and physical activity were somewhat mixed, with three studies reporting weight-based victimization to negatively impact physical activity, while three studies report no significant impact. Qualitative studies reported experiences of teasing and bullying by peers, humiliation, and feelings of insecurity about appearance as barriers to engagement in physical activity.

**Conclusion:**

The empirical evidence on the links between weight-based victimization and physical activity among community-based or primary-health care samples of adolescents who are overweight or obese is sparse. The results revealed a high presence of weight-based victimization in physical activity which seems to act as barriers for activity engagement. There is a need for more research to advance the understanding of the links between weight-based victimization and physical activity among community-based or primary-health care samples of adolescents who are overweight or obese.

## Background

Overweight and obesity have become among the world's greatest public health challenges (Lobstein et al., 2004). The World Health Organization (WHO) has recommended global, regional, and local actions to prevent the obesity epidemic from evolving further (WHO, 2012). Physical activity and exercise play an important role in weight management programs for children and adolescents with overweight and obesity by conferring several health benefits: improved physical fitness, cardiometabolic health, bone health, cognitive outcomes, mental health, reduced adiposity (Bull et al., 2020), and reduced body fat percentage (Kelley and Kelley, 2013). The WHO Guidelines on physical activity and sedentary behavior recommend that children and adolescents (5–17 years) do at least an average of 60 min/day of moderate to vigorous physical activity (Bull et al., 2020). Many adolescents do not reach the WHO's recommended level of 60 min of moderate to vigorous physical activity daily (Bull et al., 2020; Van Sluijs et al., 2021). In addition, research has found that adolescents who are overweight or obese tend to participate in less physical activity than adolescents of average weight (Olds et al., 2011; Cooper et al., 2015). Victimization is one factor that seems to have a negative impact on the prevention and treatment of overweight and obesity, and it might negatively impact engagement in physical activity (Stankov et al., 2012; Puhl and Suh, 2015; Puhl and Lessard, 2020).

Weight-based victimization and weight stigma are closely linked and highly prevalent among youth living with overweight or obesity (Lachal et al., 2013; Rees et al., 2014; Pont et al., 2017; Himmelstein and Puhl, 2019; Puhl and Lessard, 2020). Weight stigma in youth occurs most frequently in the forms of teasing and bullying about body weight (Puhl and Lessard, 2020). When referring to weight-based victimization in this scoping review, we refer to pervasive bullying and victimization as overt or relational (e.g., teasing, ignoring, excluding) forms of aggression and stigmatization toward adolescents because they are overweight or obese (Puhl and Latner, 2007; Gray et al., 2009), as weight-based victimization also is reported among some average weight adolescents (Neumark-Sztainer et al., 2002; Eisenberg et al., 2003; Frisén et al., 2009; Holubcikova et al., 2015). Experiences of weight-based bullying are found to be related to psychosocial problems, including lower motivation for physical activity, higher avoidance and emotional coping strategies, low self-esteem, and academic difficulties (Pont et al., 2017; Thompson et al., 2020). Evidence from a review of quantitative research on the health consequences of weight stigma (bullying and victimization) suggests that adolescents are highly vulnerable to weight-based victimization in physical activity settings, which might negatively impact their attitudes toward and engagement in physical activity (Puhl and Suh, 2015). A theoretical framework proposed by Salvy et al. (2012) described the association between overweight/obesity and physical activity and the negative role that peers can play in these associations. Peer social context, including the presence or absence of peer adversity and social isolation, was proposed to mediate the negative association between body weight status and physical activity. A recent narrative review by Puhl and Lessard (2020) examined the prevalence and sources of weight-based victimization targeting youth, and indicated that the consequences of these stigmatizing experiences weakens weight-related health and consequently might contribute to obesity and weight gain. According to the review, recent quantitative research examining specifically the links between weight-based victimization and physical activity in adolescents seems to be scarce and has documented somewhat mixed findings, which indicates a need to determine the extent to which weight-based victimization affects physical activity in adolescents (Puhl and Lessard, 2020).

Some studies and reviews have examined weight-based victimization among adolescents with overweight or obesity and the health consequences of weight stigma (Puhl and Suh, 2015), the different factors affecting physical activity, the effects of peers on adolescents' physical activity (Salvy et al., 2012), and the barriers to physical activity (Stankov et al., 2012). Nevertheless, these studies were conducted mainly in clinical or specialized medical treatment settings, and therefore not in the settings where most adolescents live their everyday lives.

Globally, there is an increasing awareness that weight stigma must be addressed in the treatment of overweight and obesity (Rubino et al., 2020). Specific to the focus on recommendations of increased physical activity as one of the target lifestyle determinants in the interventions offered when negative weight development is detected in primary health-care settings, weight-based victimization is relevant, as adolescents who are overweight or obese tend to be at high risk of victimization (Puhl et al., 2011; Puhl and Lessard, 2020). Also, longitudinal studies have shown that experiencing weight stigma predicts increased weight gain and obesity over time, regardless of age, baseline BMI, ethnicity, and socioeconomic factors (Puhl et al., 2017). Considering the global prevalence of overweight and obesity and the high level of inactivity among these youngsters, efforts aimed at understanding how different pertinent factors, such as weight-based victimization and stigma, may influence physical activity engagement are of high importance. Based on the abovementioned current state of knowledge, this scoping review intends to broadly identify the nature and extent of research evidence on the links between weight-based victimization and physical activity among community-based and primary health care samples of adolescents who are overweight or obese, i.e., adolescents who are not in clinical or specialist health care follow-up.

A scoping review may facilitate a more comprehensive understanding of the links between these two factors among adolescents who are overweight or obese, because it allows for a broader conceptual scope of the literature (Arksey and O'Malley, 2005) and can stimulate new research questions that can further advance research in this area (Peterson et al., 2017). The present review seeks to obtain quantitative and qualitative data to examine the nature of the literature on links between weight-based victimization and physical activity and to discuss the implications for the primary health-care follow-up of these adolescents.

## Review Objective

This review aims to identify and summarize the available published research on the links between weight-based victimization and physical activity among community-based or primary health-care samples of adolescents who are overweight or obese and to identify any existing gaps in the current state of knowledge. The broad view of this scoping review is due to the complexity of the phenomenon studied and will include the logic of both aggregating and configuring research findings (Gough et al., 2012).

## Methods

We conducted a scoping literature review guided by Arksey and O'Malley's five-step methodological framework: (1) identifying the research question, (2) identifying relevant studies, (3) study selection, (4) charting the data, and (5) collating, summarizing, and reporting the results (Arksey and O'Malley, 2005).

Prior to the database searches, we conducted preliminary searches and read previous research to screen studies for frequently used words to determine search words. We also checked different databases for key studies, which were subsequently used to inform the selection of databases for our searches. Several methods were utilized to enhance the review's validity, including using multiple researchers in the development of search words and inclusion and exclusion criteria, in performing searches, and in the selection and analysis of papers. Additionally, a senior librarian supervised the literature searches, advised on which databases were most likely to produce the type of studies we were seeking, and devised an initial search strategy, which was later refined in light of early results. This manuscript has been prepared adhering to the PRISMA extension for scoping reviews (PRISMA-ScR; Additional File 1) (Tricco et al., 2018).

### Identifying the Research Question

This review aims to identify and describe *links between weight-based victimization and physical activity among community-based or primary-health care samples of adolescents who are overweight or obese*.

The following research questions guided the objective:

1. What is the current state of knowledge about the links between weight-based victimization and physical activity among community-based or primary health-care samples of adolescents who are overweight or obese?
2. What are the research focus and findings of the presented studies on links between weight-based victimization and physical activity?

The first question aims to present an overview and a map of the studies on links between weight-based victimization and physical activity by providing details about variables, including author details, year of publication, country of origin, methodological paradigm and methods for data collection, and purpose of research. The second research question summarizes and disseminates the different approaches and findings that relate to links between weight-based victimization and physical activity among community-based or primary health care samples of adolescents with overweight or obesity in order to discuss implications for the follow-up of these adolescents and direction for future research.

The population of interest for this study consists of community-based or primary-health-care samples of adolescents who are overweight or obese—that is, who receive only follow-up in primary health care (mandatory school health service) or no follow-up at all. The investigated population should not be part of medical or clinical treatments or study interventions with the purpose of increasing engagement in physical activity.

### Identifying Relevant Studies

The search terms were categorized into four dimensions according to the review's objective. The first dimension was related to the population of interest (adolescents aged 13–18 years), the second to the categorization of the adolescents as overweight or obese, the third to experiences of weight-based victimization, and the fourth to physical activity. Search terms within the same category were separated by the Boolean OR operator, and dimensions were separated by the Boolean AND operator, as shown in Table 1.

**Table 1:** Search terms.

	**Adolescent (OR)**	**Weight (OR)**	**Stigma (OR)**	**Physical activity (OR)**
Search words (AND)	Adolescen* Youth Teen* Young* Child*	Overweight Obes* BMI “body mass” “body weight” Fat* weight	Stigma* Victim* Teas* Bully* Criticism “Peer victim*” Bias	“physical activ*” “Physical education” Sport “Health behav*” “physical exercise”

*Any word that begins with the root/stem of the word truncated by the asterisk.

To focus the scope of the review, inclusion and exclusion criteria were developed based on the review's objective (Table 2). The health-care context was set to primary health care (or nothing) to yield studies with a focus on adolescents not participating specifically in study interventions for overweight or obesity or in medical or clinical treatments. We decided to omit these populations because of the direct and indirect effects being in a specialist care context and study interventions can have on physical activity participation. Studies that were not explicitly focused on physical activity or sports behaviors were excluded, as were studies focused on fitness outcomes or motor skills. Other inclusion criteria included peer-reviewed published articles written in English on populations aged 13–18 years categorized as overweight or obese according to age and gender specific percentiles based on specified cut-off values, i.e., Iso-BMI ≥ 25 (Cole et al., 2000). Studies on populations suffering from other diseases or disabilities were excluded. We also omitted papers that did not report the relationship between weight-based victimization and physical activity in numerical or textual ways. Quantitative, qualitative, and mixed-methods studies were eligible to be included, with the purpose of considering different aspects of the links between weight-based victimization and physical activity. Due to the close link between weight-based stigma and weight-based victimization, we also included stigma in our search terms. A search without this term would narrow the result, as victimization was not always present in abstract, key words or title of relevant publications.

**Table 2:** Inclusion and exclusion criteria.

	**Inclusion**	**Exclusion**
Database	PubMed, Web of Science, Cinahl, SportDiscuss	Others
Publications	Peer-reviewed published articles	Non-peer reviewed articles, conference papers, reports, editorials, dissertations, books and book-chapters, gray literature
Topic	Studies with focus on adolescents with overweight and obesity and links between weight-based victimization and physical activity	Articles focusing on other aspects Bullying not categorized as weight-related
Population	25 ≤ Iso-BMI ≥ 25 13–18 years	Iso-BMI <25 <13 years 18 years Other diseases/diagnoses
Health care context	Community-based Primary health care School health service Healthy life centers None	Specialist health services/ secondary or tertiary care Study interventions Weight loss treatment Clinical and medical centers
Language	English	Other language

The following databases were searched to identify potentially relevant literature: PubMed, Web of science, SportDiscuss, and Cinahl. In addition, reference list searches were performed in all included studies and in reviews found through database searches to ensure relevant studies had been included in the scoping exercise (Arksey and O'Malley, 2005). This process did not yield further references. We also contacted authors to identify additional sources, but full-text screening did not produce additional references. The final search results were exported into EndNote, and duplicates were removed.

The final search strategy for PubMed can be found in Additional File 2.

### Charting the Data

Data from all included studies were charted based on authors, publication year, study aim, country of origin, research design, methodology, study population, sample size, and a summary of the key findings related to the review question (Table 3). Both reviewers, first and last author (IBS and KLH), charted the data; disagreements were solved by consensus and discussions with other reviewers (FOB and RJK) if needed.

**Table 3:** Charting of data.

**Study number/** **country/** **reference**	**Aim/** **Fit of study**	**Research design** **and analysis**	**Methodology**	**Population** **characteristics/** **health care** **setting/** **sample size**	**Key findings**
S1 Taiwan/UK Chen et al. (2010)	Examined the physical activity, attitudes toward obesity, and ideal body images of overweight/obese girls from Taiwan	Individual semi-structured interviews. Analysis: Based on the Consensual Qualitative Research approach	Qualitative	Age 13–16 years (*N* = 13), only overweight or obese girls	The major barriers to physical activity included a feeling of insecurity about appearance. The girls reported body consciousness and concerns about boys' weight-related teasing while doing physical activity, as boys' teasing was identified as the most frequent and strongest factor in relation to the pressure to be thin for the overweight/obese girls
S2 US Hand et al. (2017)	Examined weight stigma in adolescents in light of the Identity Threat Model of Stigma	Cross-sectional, multivariable, correlational design Surveys Self-reports Statistical analysis: Measures of central tendency, linear regression, and path analysis	Quantitative	Age 14–18 years (N = 302, female: 72%, overweight: 13.2%, obese: 13.9%)	IAT (weight stigma) did not significantly predict physical activity as hypothesized or as modeled in the Identity Threat Model. IAT, stress, and coping did not have an effect on physical activity. A proportion of 94% of the sample reported a positive IAT score, indicating weight stigma or bias in an overwhelming majority of the sample.
S3 US Himmelstein et al. (2019)	Examined the relationship between weight-based victimization and maladaptive eating behaviors, dieting, and weight-related health	Cross-sectional design Survey Self-reports Statistical analysis: Linear regression	Quantitative	Mean age 15.6 years (*N* = 9,838, overweight or obese, *N* = 37.2%) National sample of Sexual or Gender minority adolescents	Frequency of weight-based victimization at school was associated with more physical activity but also with more reasons to avoid exercise. Family teasing was associated with lower levels of physical activity and more reasons to avoid exercise, while teasing from peers was associated only with exercise avoidance. BMI percentile and gender identity were consistently associated with less physical activity (exception: non-binary) and more exercise avoidance. BMI percentile was consistently associated with poor weight-related health across all models except trouble sleeping. This study examined weight-related victimization but not specifically in the physical activity context.
S4 UK Lewis et al. (2014)	Explored the experiences of overweight and obese children and young people who had successfully increased their activity levels	Semi-structured interviews Analysis: Template analysis	Qualitative	*N* = 58 (aged 6–11, *N* = 39; aged 12–16, *N* = 19) All children had a BMI above the 91st percentile (overweight) or above the 98th percentile (obese) for their age and gender. Children enrolled in a community-based program consisting of activities to help children and young people who were overweight and obese, encourage weight loss, and improve morale.	Older children described how they did not feel bullied at the community activity sessions compared with school and felt it was a collective experience, being with other overweight young people, and that the instructors were more respectful toward them. Their success in the scheme led them to feeling more capable. They described how they were more confident to be active in front of their “normal-weight” peers, as well as their overweight peers.
S5 US Li and Rukavina (2012)	Examined (1) the nature and occurring contexts of weight-related teasing in urban PE programs and (2) the psychological implications of weight-related teasing	Descriptive case study approach Semi-structured in-depth interviews Analysis: Inductive analysis and constant comparisons	Qualitative	Age 11–19 years (*N* = 47) BMI greater than the 85th percentile for age. Recruited from a large urban school district.	Most overweight or obese adolescents (68%) experienced negative assumptions in PE before performing a task: verbal prejudgments about being overweight or about obese students' capabilities. The assumptions were related to personality, social skills, and athletic capabilities. The participants reported peers having negative assumptions about their fitness and game-playing capabilities. As a consequence, the adolescents indicated that they were commonly excluded or one of the last to be selected for the team and were discouraged from being actively engaged in PE. Most participants (64%) reported being teased in PE. Students who were overweight or obese felt uncomfortable participating in PE due to social comparison in the public display of skill in fitness activities. They reported feeling self-conscious when classmates made comments about their weight. A proportion of 36% of participants who were overweight or obese reported that they did not experience weight-related teasing in PE. Adolescents who were overweight mentioned feeling hurt, sad, isolated, unappreciated, or even depressed. Overweight adolescents felt inferior to their peers when participating in fitness activities because they ran slower or did fewer push-ups than peers.
S6 US Li et al. (2012)	Explored the coping mechanisms that adolescents perceived to be overweight or obese used to cope with weight-related teasing in school PA	In-depth interviews with all participants, one focus group interview with six students Analysis: Inductive analysis and constant comparisons	Qualitative	Age 11–19 years (*N* = 30, girls: 20) Body mass indexes larger than the 85th percentile for age. Enrolled in PA at the time of interviews or in the previous year. Urban city school district in the southern US.	To compensate for weight stigma, individuals perceived to be overweight or obese can do things to increase their likability or work hard to improve other's perceptions of their abilities or skills. Two adolescents tried to improve their peers' perception of their abilities or skills by working hard to get better at sports or games.
S7 Canada Maïano et al. (2018)	Two-fold objective: (1) replicated and extended previous research on adolescents with overweight and obesity by examining the relations between perceived weight-related victimization in school-based PA and	Cross-sectional design Questionnaire Self-reports Statistical analysis: Factor analysis, correlation analysis	Quantitative	Age 14–18 years (*N* = 144, girls: 69, overweight: 76.4%, obese: 23.6%)	Adolescents with overweight and obesity who were more frequently exposed to weight-related victimization tended to report lower levels of perceived physical abilities, which in turn were related to lower levels perceived PE performance, as well as to lower levels of physical activity involvement outside the school setting. The results appeared unchanged by characteristics such as sex and age; it seems that perceived
	students' perceived PE performance or involvement in PA outside school and (2) investigated the indirect role of perceived physical abilities and fear of enacted stigma on these relations				physical abilities are likely to be harmful to the perceived PE performance and participation in physical activity outside of school of adolescents of all ages and of both sexes. Although exposure to weight-related victimization was found to predict internalization of weight stigma among youth with overweight and obesity, such internalization did not predict their perceived PE performance levels or their involvement in physical activity outside school setting. These results thus suggest that even though perceived weight-related victimization is positively related to the internalization of weight stigmatization, the resulting fear of being further stigmatized does not translate to lower perceived PE performance or to a greater tendency to avoid physical activity outside school.
S8 US Puhl and Luedicke (2012)	Examined the ways that adolescents cope with experiences of weight-based victimization at school	Cross-sectional design Questionnaires Self-reports Statistical analysis: Ordered and binary logistic regression, structural equation modeling techniques	Quantitative	Students in grades 9–12 (*N* = 394, overweight: 18%, obese: 17%) who reported having experienced weight-based victimization	This paper examined weight-based victimization in general, although the participants were asked how often they had experienced weight-based victimization at eight different locations on the school campus, including the gymnasium and athletic field. The participants were also asked how often they used 28 different coping strategies in response to experiences of weight-based victimization at school; some of the avoidance strategies were “I avoided doing physical activities” and “I avoided going to gym class.” Weight-based teasing during gym class was strongly related to avoidance coping strategies among girls (regardless of BMI status). The number of teasing incidents had a direct effect on avoidance coping strategies for girls but not boys. The more that adolescents reported negative affect in response to weight-based victimization, the more they reported coping with avoidance strategies (e.g., avoiding physical activity) and using maladaptive coping strategies that involved increased food consumption and binge eating. An unexpected finding in this study is the high percentage of youth at a healthy weight who reported experiencing weight victimization and whose responses to victimization
					were similar to those of their heavier peers. The findings of the study suggest that overweight girls are especially vulnerable to negative emotions resulting from weight-based victimization that occurs in the context of engaging in physical activity and that this may lead to avoiding future physical activity in an attempt to prevent additional victimization.
S9 UK Reece et al. (2016)	Explored the experiences of obese adolescents and their perspectives on obesity treatments	One-to-one interviews, small focus groups with overweight and obese young people. Analysis: Framework method	Qualitative	Age 11–16 years (*N* = 12), attending a community weight management intervention (adhering to NICE guidance and delivered by multidisciplinary teams in community venues)	Feelings of dissatisfaction with body image and physical appearance and negative experiences of being bullied were described as reasons for wanting to change. Once engaged in treatment, there was unequivocal agreement among the young people that the experience of interacting with peers in a socially supportive context was enjoyable and conducive to losing weight. It seems likely that this may have been one of the first times that these young people had been able to physically interact with peers in a safe and supportive environment, where they were not stigmatized or shamed for being overweight or obese. The participants described treatment groups as safe and supportive environments where they were not stigmatized and where they could be physically active.
S10 Canada Stearns et al. (2017)	Empirically tested whether negative peer experiences led adolescents with overweight and obesity to be less active and engage in more sitting-related behaviors	Cross-sectional design. Questionnaire Self-report Statistical analysis: Multilevel path analysis	Quantitative	Students in grades 9–12 attending 43 secondary schools in Ontario, Canada (*N* = 24,173) 19% of females and 32% of males were overweight or obese, and 21% of females and 15% of males had been victimized at least once during the last 30 days.	While females' weight status was positively associated with peer victimization, peer victimization was positively associated with moderate to vigorous physical activity. Results indicates that females with overweight/obesity participated in 0.074 fewer hours per day (or 4 min per day) of moderate to vigorous physical activity than females who were not overweight. The indirect effect of peer victimization was significant. Specifically, females who were overweight/obese had 0.015 additional hours per day (or 1 min per day) of moderate to vigorous physical activity compared to females who were not overweight, which could be attributed to increased peer victimization. Females with overweight/obesity did engage in less moderate to vigorous physical activity and were more likely to have been victimized compared to adolescents who were not overweight; however, those who were victimized tended to
					perform more moderate to vigorous physical activity. Males' weight status was positively associated with peer victimization. Controlling for weight status, peer victimization was not associated with moderate to vigorous physical activity for males. The pathway between weight status and moderate to vigorous physical activity was significant for males. The indirect effect of peer victimization was not significant.
S11 Brazil Watanabe et al. (2017)	Determined the association between weight-teasing and physical activity in students from public schools of Curitiba, Paraná	Cross-sectional design Questionnaires Self-reports Physical activity level evaluated using an accelerometer Statistical analysis: Correlation	Quantitative	Age 13.2 ± 0.8 years (*N* = 95), overweight = 25 (26.3%), enrolled in the public-school system	Participants who were overweight had higher prevalence of weight-teasing and teasing during physical activity for the total sample and for males. The correlations between the continuous variables of physical activity and the variables of weight-teasing were non-significant in both sexes. Association between weight teasing and moderate to vigorous physical activity were weak and non-significant. The same was observed for weight-teasing during physical activity. Concerning teasing that occurred specifically in the context of physical activity, the values found were 10% for normal weight and 44% for overweight participants.
S12 Norway Øen et al. (2018)	Gained an in-depth understanding of the perspectives and life experiences of adolescents living with obesity	Descriptive, qualitative approach with individual qualitative interviews Analysis: Qualitative content analysis	Qualitative	Age 12–15 years (*N* = 5, girls = 4), Iso-BMI > 30, had in some way been in contact with health-care providers about their obesity	The participants feared being discredited in social settings generally, and during physical activities in particular. They experienced a lack of social support in situations involving organized sports, and all but one had experienced difficult situations related to their weight and appearance. Some experienced bullying, including being insulted, experiences with a lasting effect on their emotional state and motivation to make behavioral changes. The participants in the study suggested that being in a group with other adolescents suffering from obesity was a preferred arena to share experiences, obtain peer support, and perhaps participate in pleasant activities together. The respondents appeared to be vulnerable and seemed to have few resources to handle teasing, bullying, and stigmatizing or to succeed in health-promoting activities.

### Collating, Summarizing, and Reporting Results

The data from the included studies were put into a table and analyzed descriptively and numerically in terms of the extent and nature of the studies. According to scoping review guidelines (Arksey and O'Malley, 2005), the findings were discussed considering the objective of this review and implications for research and practice. In line with the PRISMA-ScR (PRISMA Extension for Scoping Reviews) checklist (Tricco et al., 2018), no studies were evaluated for quality; thus, reporting in this review was based on a direct presentation of the results from the included studies.

To ensure consistency between the two reviewers, both IBS and KLH evaluated the title, abstract, and full text of each of the studies included in this review. Disagreement on study selection and data extraction was solved by consensus and discussions with other reviewers when needed (Tricco et al., 2018).

## Results

**Figure 1:**
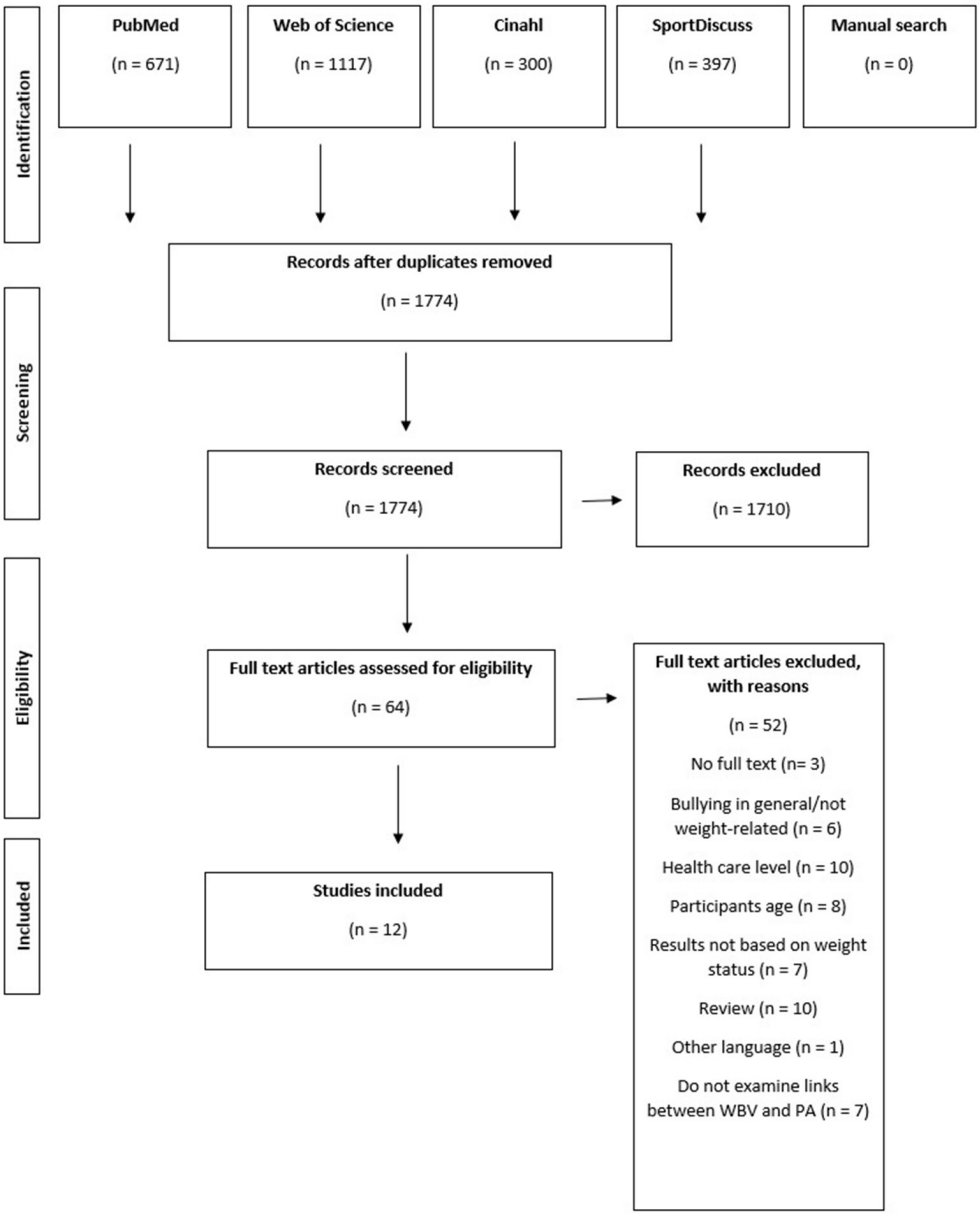
Flowchart diagram of the screening and selection procedure.

After removing duplicates, 1,774 citations were identified from searches of electronic databases and review article references. Based on the title and the abstract, 1,710 were excluded, with 64 full-text articles retrieved and assessed for eligibility. Of these, 52 were excluded for the reasons shown in Figure 1. The remaining 12 studies were considered eligible for this review (Table 3). This section provides a descriptive overview of the breadth of the literature and a numerical analysis of the extent and nature of the studies using tables, followed by a textual presentation of the findings relevant to the objective of this scoping review.

### Study Selection

Figure 1 shows the process of selecting studies for the scoping review according to the PRISMA-ScR (PRISMA Extension for Scoping Reviews) checklist (Tricco et al., 2018), detailing the decision process. The flowchart includes search results, selection process results, additions from reference list searching, and the final number of included sources. The database searches were performed during the period 23 Nov 2020–02 Dec 2020, with the last database search conducted on 02 Dec 2020.

### General Characteristics of the Included Studies

#### Distribution by Year of Publication

Of the studies included in this scoping review, the first article that takes links between weight-based victimization and physical activity into account was published in 2010 (Chen et al., 2010). The distribution of published articles on this topic is about one in a year. However, two were published in 2012, two in 2018, and three in 2017. The latest article identified for this scoping review was published in 2019.

#### Distribution by Country

Nearly half of the included 12 studies were conducted in the US, three in the UK, and two in Canada. The last two were conducted in Brazil and Norway.

#### Methodological Paradigm and Methods for Data Collection

The distribution of quantitative and qualitative studies was six of each. None of the included studies used a mixed-method approach.

The qualitative studies used individual interviews, semi-structured interviews, or in-depth interviews (Chen et al., 2010; Li and Rukavina, 2012; Li et al., 2012; Lewis et al., 2014; Reece et al., 2016; Øen et al., 2018). Two of these studies included focus group interviews (Li et al., 2012; Reece et al., 2016). All the papers in the quantitative paradigm were cross-sectional and used surveys and questionnaires (Puhl and Luedicke, 2012; Hand et al., 2017; Stearns et al., 2017; Watanabe et al., 2017; Maïano et al., 2018; Himmelstein et al., 2019). Self-reports of demographic and anthropometric characteristics were reported in five quantitative studies (Puhl and Luedicke, 2012; Hand et al., 2017; Stearns et al., 2017; Maïano et al., 2018; Himmelstein et al., 2019). One study used self-reports of physical education (PE) performance (Maïano et al., 2018), and another self-reports of moderate to vigorous physical activity and screen time (Stearns et al., 2017). Among the quantitative studies, one assessed physical activity level using accelerometers (ActiGraphGT3X) (Watanabe et al., 2017).

#### Purpose of the Research

The general aims of the quantitative studies included in this scoping review centered on examining the relationship between weight-based victimization, peer victimization or stigma and physical activity in itself or as one of several factors with a role in weight-related health, not especially directed toward adolescents who are overweight or obese as these were rather sub-groups of the samples in the included quantitative studies (see Table 3). Two quantitative studies do not mention this relationship in the aim of the research but report on the association in the findings (Puhl and Luedicke, 2012; Hand et al., 2017). All quantitative studies included overweight or obese adolescents in accordance with our inclusion criteria. However, five (Puhl and Luedicke, 2012; Hand et al., 2017; Stearns et al., 2017; Watanabe et al., 2017; Himmelstein et al., 2019) of the six studies included participants in other weight categories as well. Only the study by Maiano, Lepage, Aimé and Morin (Maïano et al., 2018) had a sample of exclusively adolescents who were overweight or obese. This study was also the only quantitative study with a research purpose exclusively directed to this group of adolescents.

In the qualitative paradigm, the studies were directed more toward broader aspects of life as an adolescent with overweight or obesity and reported on links between weight-based victimization and physical activity because of adolescents' sharing experiences through the interviews. All qualitative studies included exclusively participants who were overweight or obese (Table 3). Four of the research articles mentioned physical activity in or outside of the school setting and a link to the everyday life experiences of these adolescents in their aims (Chen et al., 2010; Li and Rukavina, 2012; Li et al., 2012; Lewis et al., 2014). The two other qualitative studies explored experiences related to obesity treatment (Reece et al., 2016) and the perspectives and life experiences of adolescents with overweight or obesity (Øen et al., 2018). The two latter studies reported on experiences of weight-based victimization related to physical activity in their results. The work of Chen et al. (2010) centered on understanding body image and physical activity for weight control among adolescent girls from Taiwan who are overweight or obese.

One study examined associations only in girls who were overweight or obese (Chen et al., 2010), and another in sexual and gender minority adolescents (Himmelstein et al., 2019).

### Overview of Findings Relevant to the Objective

Table 3 summarizes the general characteristics and results relevant to the objective of this scoping review. Due to the different approaches of the qualitative and quantitative studies contributing to different perspectives regarding the objective of this scoping review, the main results from studies were grouped under these two paradigms and are textually described in the following two paragraphs.

### Findings of Quantitative Studies

The studies in the quantitative paradigm showed that there is high frequency of weight-based victimization among adolescents who are overweight or obese. However, the overall findings of these studies are somewhat mixed due to the different aims and approaches of the studies; for example one study reported that peer victimization was positively related to moderate to vigorous physical activity for girls who are overweight or obese (Stearns et al., 2017), another revealed that weight stigma did not affect physical activity (Hand et al., 2017), and yet another study found that weight-based teasing during gym class was among girls strongly related to avoidance coping strategies (Puhl and Luedicke, 2012).

In addition, some of the included studies contribute with a high portion of findings relevant to the aim of this scoping review, while others serve rather small contributions. The main findings of the quantitative studies are organized in three different themes: effect on physical activity, the importance of gender, and the role of context.

#### The Effect of Weight-Based Victimization and Stigma on Physical Activity

The effect of weight-based victimization and stigma on physical activity is inconclusive among the studies as half of the studies report no effect, while the other half report some effect on different variables.

Although the study by Hand et al. (2017) report that weight stigma (verbal teasing, physical and cyber bullying, and social exclusion) is prevalent in an overwhelming majority of the sample regardless of weight status, weight stigma did not have an effect on physical activity. In line with this, also Stearns et al. (2017) report that peer victimization did not mediate a negative association between body weight status and moderate- to vigorous physical activity. Watanabe et al. (2017) determined an association between weight teasing and physical activity. In this study, the group of overweight adolescents experienced more weight-teasing and teasing during physical activity compared to those who were not overweight in the total sample and in the male sample. Nevertheless, the association between weight-teasing and moderate to vigorous physical activity was weak and non-significant, and this was also the case for weight teasing during physical activity.

BMI percentile was associated with less physical activity and more avoidance of exercise among a sample of sexual and gender minority adolescents were over half of the sample reported experiences of weight-based victimization, in the study by Himmelstein et al. (2019). In this study weight-based victimization contributed to an increased likelihood of many adverse health behaviors, such as lower physical activity. These health consequences occurred regardless of the adolescents' age, ethnicity, BMI, sexual identity, or gender identity. Puhl and Luedicke (2012) report that weight-based victimization was a frequent experience for adolescents in all weight categories, and strategies of avoidance of physical activity were likely to be used among adolescents who reported negative affect in response to weight-based victimization. The more the adolescents' response to being victimized generated a negative effect, the more they also used the coping strategy of avoidance of physical activity. Yet another study, Maïano et al. (2018), reported that weight-based victimization among adolescents who were overweight or obese had a negative impact on physical activity variables; perceived weight-based victimization negatively predicted perceived physical abilities. Fear of enacted stigma on the other hand, was not a significant predictor of involvement in physical activity.

#### Gender

Some studies also report mixed results overall (e.g., when using more than one measure for weight-based victimization or in sub-analyses by gender or weight). Especially regarding gender, four of the studies were able to report especially on this due to the sample characteristics (Puhl and Luedicke, 2012; Stearns et al., 2017; Watanabe et al., 2017; Maïano et al., 2018). Stearns et al. (2017) found that females with overweight or obesity engaged less in moderate to vigorous physical activity compared to females who were not overweight and were also more likely to be victimized, but peer victimization was positively associated with moderate- to vigorous physical activity among girls. For males, on the other hand, the indirect effect on moderate to vigorous physical activity through peer victimization was not significant when controlling for weight status. In the study by Puhl and Luedicke (2012) they reported gender differences to emerge in how they react to experiences of weight-based victimization. Girls attributed more negative emotions to weight-based teasing than boys, and they also reported more than boys that weight teasing made them feel sad, depressed, worse about themselves, and having bad feelings about their body. Overweight girls were especially vulnerable to negative emotions resulting from weight-based victimization that occurs in the context of engaging in physical activity and this may lead to avoidance of future physical activity in an attempt to prevent additional victimization. However, although girls are especially prone to negative affect in their reactions to weight-based victimization, both boys and girls who reported negative affect in response to weight-based victimization were more likely to use coping strategies of avoidance of physical activity. On the opposite, there were no significant correlations between the variables of physical activity and weight-teasing in either sex in the study by Watanabe et al. (2017). In the study by Maïano et al. (2018) the association between weight-based victimization and physical activity were unchanged by gender and showed that in the relationship between perceived weight-based victimization and perceived physical education performance or involvement in physical activity outside school, the perceived physical abilities were found to have a significant and negative indirect role. This was also the only study including exclusively a sample of adolescents who were overweight and obese.

#### The Physical Activity Context

Two studies, Maïano et al. (2018) and Puhl and Luedicke (2012), reported on differences regarding the physical activity context. When investigating the relationship between the frequency of perceived weight-based victimization among adolescents who are overweight or obese and physical activity, Maïano et al. (2018) indicated that there might be a difference in engaging in physical activity in and outside the school setting. Perceived experiences of weight-based victimization in the school setting did not directly predict engagement in physical activity outside the school context among these youngsters, indicating that the two settings are perceived differently for physical activity engagement. Internalization of weight stigma among adolescents did not predict levels of physical activity engagement outside school settings. The same study found that adolescents who experience weight-based victimization more frequently tended to report their perceived physical abilities at a lower level, which was further related to lower levels of engagement in physical activity outside the school setting.

Puhl and Luedicke (2012) examined weight-based victimization in the school setting and found that adolescents who reported emotional distress in response to weight bullying were more likely to avoid physical activity as one among other coping strategies, especially if teased in gym class (Puhl and Luedicke, 2012).

### Findings of Qualitative Studies

All qualitative studies also showed a high presence of weight-based victimization in physical activity in various settings among adolescents who were overweight or obese. Due to the different aims and approaches of the qualitative studies included in this scoping review, the results contribute different perspectives on the nature of weight-based victimization and physical activity experienced by adolescents who are overweight or obese. Nevertheless, when summarizing the results some common themes emerged from the studies: relational victimization, physical activity with similar others, and reactions to and coping with victimization.

#### Relational Victimization

Qualitative studies reported that the barriers to physical activity included experiences of teasing and bullying by peers, humiliation, and feelings of insecurity about appearance in one way or another (Chen et al., 2010; Li and Rukavina, 2012; Lewis et al., 2014; Øen et al., 2018). Exploring weight-based teasing in urban physical education programs, Li and Rukavina (2012) found that most adolescents with overweight or obesity experienced negative assumptions in physical education classes. As a consequence of experiencing negative assumptions from peers, they were commonly excluded, were the last teammate to get selected, and felt discouraged from being actively engaged. The study showed that adolescents with overweight or obesity were uncomfortable actively taking part in physical education because of the social comparison in the public display of skills in fitness testing and activities. An interesting finding in this study was that not all students who were overweight or obese were teased in physical education classes for a variety of reasons (e.g., family-oriented school environment, other students had larger body sizes, good personality being outgoing and cool, talented in academics or athletics, teasers were afraid of them or being more active than peers), which indicates that there might be important protective factors against teasing experiences. A study by Øen et al. (2018) indicated that adolescents were particularly afraid of being discredited during physical activities. The findings reflect the perspectives and life experiences of adolescents living with obesity. The participants described experiencing a lack of social support in organized sports and difficult situations because of their weight and appearance. Some adolescents experienced bullying and being insulted, which might have affected their motivation to make behavioral changes. Insecurity about appearance was the major barrier to physical activity in the study by Chen et al. (2010), and being teased by boys while doing physical activity was identified as the most pertinent factor in the relation to be thin for girls. Also, in the study by Lewis et al. (2014) being bullied was described as a common experience, in this study related to being physical active with “normal weight” peers.

#### Physical Activity With Similar Others

A common perspective among half of the qualitative studies was that being with other adolescents with overweight when engaging in physical activity was reported as a positive experience or something that the adolescents believed would act as a facilitator for physical activity engagement (Lewis et al., 2014; Reece et al., 2016; Øen et al., 2018). In the study by Lewis et al. (2014), children and adolescents who were overweight, obese, and physically active described activity sessions with similar children with overweight or obesity as a collective experience. By participating in such activity settings, they felt more capable and more confident to be active in front of “normal weight” peers. The participants in this study also valued the opportunity to extend their social network for physical activity by participating in such groups, and the adolescents found the sessions intrinsically rewarding. Also, in another study participants described experiences of interacting with peers in socially supportive context as enjoyable (Reece et al., 2016). For many adolescents such activity sessions seemed to be one of the first times these adolescents were able to be physically active without being stigmatized or shamed due to their weight. They distinguished these sessions from the experiences of physical activity at the school, where they felt bullied (Reece et al., 2016). Being in a group with other adolescents with shared experiences of being obese was also suggested by participants in the study by Øen et al. (2018) as a preferred arena where they also could participate in pleasant activities together.

#### Reactions to and Coping With Victimization

A study of the coping mechanisms against weight-based teasing among adolescents perceived to be overweight or obese in PE found that adolescents used self-protection, compensation, confrontation, seeking social support, avoidance/psychological disengagement, and stress-reduction strategies to cope against weight-based teasing. Some adolescents were motivated to lose weight because they were tired of negative comments. This study suggests that adolescents who are overweight or obese can do things to increase their likability or improve other's perceptions of their abilities and skills in physical activity by working hard to get better at sports or games (Li et al., 2012). Another qualitative study reported a similar perspective among participants who believed that weight loss would stop the bullying, which seemed to motivate the adolescents to change health behaviors (Reece et al., 2016). Chen et al. (2010) explored adolescent girls' perspectives, providing a preliminary understanding of body image and physical activity for this group of adolescents. Their study showed how living with overweight or obesity had an impact on beliefs, self-perceptions, and behaviors among these girls. The researchers also proposed an understanding of the complexity of life as overweight, where a major motivation for physical activity is losing weight or improving appearance, as the participants valued thinness. The girls' expressed barriers to physical activity were mainly being teased and feeling insecure about their appearance.

### Study Participants' Weight Status and Findings Considering the Topic's Sensitivity

In terms of participants' weight status, there is a difference regarding the methodological paradigm. In nearly all quantitative studies adolescents in all weight categories were included in the samples, but only studies where results specifically for adolescents who are overweight or obese are described, were included in this scoping review (see Table 3). Maïano et al. (2018) was the only quantitative study with all participants being overweight or obese, with a sample of 144 secondary school students (Maïano et al., 2018). Among the qualitative studies all included exclusively participants in the overweight or obesity category, and the number of participants ranged from 5 to 58. Table 3 provides an overview of the sample distribution in the included studies.

As the main outcome of studies including exclusively participants who were overweight or obese showed a tendency toward weight-based victimization having a negative impact on physical activity engagement, the findings were slightly different among studies with participants in all weight categories (Puhl and Luedicke, 2012; Hand et al., 2017; Stearns et al., 2017; Watanabe et al., 2017; Himmelstein et al., 2019). Several studies found that weight-based victimization is of high frequency regardless of adolescents' weight status (Puhl and Luedicke, 2012; Hand et al., 2017; Himmelstein et al., 2019), and that this does not have a great negative impact on physical activity engagement among adolescents with overweight or obesity compared to adolescents of average weight. Watanabe et al. (2017) report that the overweight group had higher prevalence of weight-teasing and teasing during physical activity for the total sample and for males, but no significant correlations were found to the variable of physical activity.

### Significant Others

Most studies reported weight-based victimization from peers (Chen et al., 2010; Li and Rukavina, 2012; Li et al., 2012; Puhl and Luedicke, 2012; Lewis et al., 2014; Reece et al., 2016; Stearns et al., 2017; Watanabe et al., 2017; Maïano et al., 2018; Øen et al., 2018; Himmelstein et al., 2019), and two explicitly mentioned victimization from family (Chen et al., 2010; Himmelstein et al., 2019). Adolescents' need for social support is pointed out in some of the included papers, with support being that the participants emphasized physical activity with other adolescents who were overweight as a way to make social connections and feel part of a group (Lewis et al., 2014; Reece et al., 2016; Øen et al., 2018).

## Discussion

This scoping review provides an overview of studies examining the links between weight-based victimization and physical activity among community-based or primary health care samples of adolescents who are overweight or obese, including both aggregative and configurative research approaches and hence different aspects of the links are reported. *Twelve* studies were identified across various life settings of adolescents with overweight and obesity: six qualitative and six quantitative studies.

The research focus of the included studies in this scoping review revealed gaps in the literature. Few studies have been performed with the links between weight-based victimization and physical activity as the primary focus of research, and there is a homogeneity of the research designs applied, which suggests that there is a need to expand methodological approaches. Further, only seven studies included exclusively participants who were overweight or obese, and among these only one study with a quantitative design were represented (Maïano et al., 2018). Also, the study selection process revealed that a large number of studies on the topic of interest for this scoping review are conducted in secondary or tertiary care, as study interventions or in clinical or medical centers. The low number of included studies in this scoping review revealed that studies carried out among community-based or primary health-care samples of adolescents who are overweight or obese are sparse.

The links between weight-based victimization and physical activity has two different approaches in this scoping review. The included studies of more aggregative nature best answered the question on how weight-based victimization affect the physical activity participation, while studies with a more configurative approach made it possible to elaborate on how adolescents experience, react to and cope with weight-based victimization in physical activity.

The main findings of both quantitative and qualitative research were that adolescents who are overweight or obese frequently experience weight-based victimization, and this overall tends to affect engagement in physical activity in a negative way. There seems to be a discrepancy regarding the reported link between weight-based victimization and physical activity depending on the participants weight status. Studies including exclusively participants who are overweight or obese document victimization to negatively impact physical activity participation in some way (Chen et al., 2010; Li and Rukavina, 2012; Li et al., 2012; Lewis et al., 2014; Reece et al., 2016; Maïano et al., 2018; Øen et al., 2018). In the studies including all weight categories, all with aggregative approaches, three studies report no association (Hand et al., 2017; Stearns et al., 2017; Watanabe et al., 2017), while two report some association (Puhl and Luedicke, 2012; Himmelstein et al., 2019). The finding might be due to the designs and methods applied for these latter studies; weight-based victimization is reported in all weight categories which might affect the result. Among studies in the qualitative paradigm, findings report weight-based victimization in physical activity to act as barriers for participation as adolescents' experience feelings of inferiority, insecurity about appearance, lack of social support and negative assumptions from peers. In the studies it is reported that the adolescents are uncomfortable taking part in physical activity and more than one study report that losing weight is a motivational factor to stop bullying, which might increase the physical activity level but not necessary in a healthy way (Chen et al., 2010; Li et al., 2012; Reece et al., 2016). Gender differences are only reported in quantitative studies and indicate in some studies that girls may react more to weight-based victimization than boys. However, a negative link between weight-based victimization and physical activity is found in both genders (Li and Rukavina, 2012; Li et al., 2012; Puhl and Luedicke, 2012; Lewis et al., 2014; Reece et al., 2016; Maïano et al., 2018; Øen et al., 2018). Also, the school setting seems to be an especially vulnerable setting for weight-based victimization for these adolescents (Puhl and Luedicke, 2012; Maïano et al., 2018; Øen et al., 2018). Another common finding in some studies is the importance of social support for engaging in physical activity (Li et al., 2012; Lewis et al., 2014; Reece et al., 2016; Øen et al., 2018).

### Methodological Paradigm and Methods for Data Collection

In the 12 included studies, there is little diversity in research methods applied. All six qualitative studies used individual interviews as a method for data generation; additionally, two studies used small focus groups (Li et al., 2012; Reece et al., 2016). All six quantitative studies used a cross-sectional design. Furthermore, all quantitative studies gathered the demographic and anthropometric characteristics of the participants via self-reports. Only one study used objective data from accelerometers to collect information on physical activity level (Table 3) (Watanabe et al., 2017).

The included studies are equally distributed in the qualitative and quantitative paradigms and contribute to different aspects of the links between weight-based victimization and physical activity among adolescents who are overweight and obese. The distinguishing features between these two paradigms become evident in the studies included in this scoping review. The quantitative studies typically addressed patterns and characteristics in the populations, while the qualitative ones generally explored how weight-based victimization occur and described how the phenomenon was experienced by the individuals (Fetters et al., 2013).

A broader understanding of the multi-dimensional nature of physical activity engagement in adolescents with overweight or obesity may require both quantitative and qualitative research methods. There might be a need to combine methods (Mixed method research) to gain a broader understanding of the phenomenon and where the strengths in one method can compensate for the weaknesses in another. None of the included studies took a mixed-methods approach, which aims to draw upon the strengths of quantitative and qualitative methods as an innovative way of addressing contemporary issues in health research (Fetters et al., 2013). While quantitative methods contribute with estimates of common patterns, characteristics, and trends in a population (aggregative approach), qualitative methods seek to explore and articulate interactions, texture, and conceptual and empirical diversity (configurative approach) (Oancea et al., 2017). Combining these methodologies could provide richer data and contribute to a deeper conceptual and contextual understanding of the phenomenon.

### Research Focus of the Included Studies

Only five included studies more directly addressed the links between weight-based victimization and physical activity among adolescents who are overweight and obese (Li and Rukavina, 2012; Li et al., 2012; Stearns et al., 2017; Watanabe et al., 2017; Maïano et al., 2018). Other studies found links between weight-based teasing and physical activity more implicitly as part of the results of, for example, “experiences of physical activity” (Chen et al., 2010) and outcomes from weight-based victimization (Himmelstein et al., 2019). In some studies, it was unclear whether weight was the reason for victimization and whether the victimization took place in the setting of physical activity; the results nonetheless describe links between weight-based victimization and physical activity. Future work should specifically focus on weight-based victimization among adolescents who are overweight or obese in physical activity settings to better understand the experiences essential to this context.

### The Links Between Weight-Based Victimization and Physical Activity

The low number of identified studies in this scoping review indicates a lack of research focusing directly on the links between weight-based victimization and engagement in physical activity among adolescents with overweight or obesity outside of medical treatment or study interventions. Overall, adolescents with overweight or obesity seem to be vulnerable to victimization, which—according to the findings of this preliminary review—might keep them from engaging in physical activity in their everyday life. All included studies based on interviews showed a high degree of congruence. They reported that experiences of weight-based victimization, in one way or another, act as barriers to engagement in physical activity.

The qualitative studies in the present review reported adolescents with overweight and obesity to perceive victimization and feelings of inferiority, insecurity of appearance, lack of social support and negative assumptions from peers as barriers to engaging in physical activity. These findings are in line with a systematic review of qualitative studies undertaken to understand the barriers to physical activity experienced by adolescents who were overweight and obese (Stankov et al., 2012). Nonetheless, this scoping review provides an opportunity to explore the associations and paths between weight-based victimization and physical activity for more extensive samples of adolescents in various settings, which shows that in-depth studies of the experiences of adolescents with overweight or obesity are coherent about how different forms of victimization and stigma act as barriers for engagement in physical activity among these adolescents. Our work agrees with a recent review by Puhl and Lessard (2020) on related topics and shows that quantitative studies of the links between weight-based victimization and physical activity document somewhat mixed findings. Still, three of the six quantitative studies reported significant links between weight-based victimization and physical activity among adolescents who were overweight and obese (Puhl and Luedicke, 2012; Maïano et al., 2018; Himmelstein et al., 2019). Nevertheless, all papers found peer victimization to be a frequent experience. The mixed findings in quantitative studies might be due to several reasons, among these, low sample specificity could be part of the explanation.

It has been previously reported that adolescents who are overweight or obese tend to participate in less physical activity than adolescents of average weight (Olds et al., 2011; Cooper et al., 2015). Nevertheless, one study reported that adolescents might be motivated to lose weight to stop negative comments from peers (Li et al., 2012), and one study found that adolescents seemed to be driven to change their health behavior by the belief that bullying would stop (Reece et al., 2016). However, when adolescents are motivated to lose weight because of teasing, they are more likely to engage in unsafe weight loss behaviors compared to adolescents motivated by improved health or sport outcomes according to findings from the National Health and Nutrition Examination Survey conducted in the US (Brown et al., 2016).

A better understanding of the links between weight-based victimization and physical activity for this group of adolescents might therefore be of importance. Few studies have focused on what adolescents emphasize in an environment to be physically active. However, three of the identified studies reported that the adolescents preferred to engage in activities with other adolescents who were overweight or obese, as such an environment was perceived as supportive and safe (Lewis et al., 2014; Reece et al., 2016; Øen et al., 2018). We suggest that future research should focus on what is vital for adolescents with overweight and obesity in an environment they perceive as supportive and safe for activity engagement.

### Study Participants' Weight Status, the Context for Physical Activity and Gender

Among the 12 included studies, 7 included exclusively participants who were overweight or obese (Chen et al., 2010; Li and Rukavina, 2012; Li et al., 2012; Lewis et al., 2014; Reece et al., 2016; Maïano et al., 2018; Øen et al., 2018). This is an indication of an important challenge for such studies. Several works have pointed out the challenges in recruiting these adolescents for participation in research because of the topic's sensitivity (Li and Rukavina, 2012; Li et al., 2012; Øen et al., 2018). Especially in the quantitative studies participants of all weight categories were included, therefore it might be of importance for future research to conduct research with aggregative approach on more specific samples of adolescents who are overweight or obese.

All six qualitative studies comprising exclusively adolescents with overweight or obesity reported barriers to participating in physical activity due to different experiences with victimization, such as teasing and negative assumptions from peers and insecurity about appearance. Among studies with participants representing all weight categories, the results are slightly different. Previous studies found that weight-based victimization is highly evident also among people who are not overweight (Frisén et al., 2009; Holubcikova et al., 2015), and this tendency might have led to findings were adolescents who are overweight or obese do not significantly differ regarding the impact on physical activity engagement compared to adolescents of other weight categories. This may for example be the case in the included study by Hand et al. (2017) which report that although the majority of the sample was in the normal weight category, “about the right weight” was perceived and reported by less than half of the sample. In the study by Puhl and Luedicke (2012) they elaborate on this and also find that a high percentage of youth at average weight report experiencing weight-victimization, and whose responses correspond to their heavier peers.

In the quantitative study comprising exclusively participants who were overweight or obese, the authors reported that perceived weight-based victimization experiences occurring in school settings do not directly predict the levels of involvement in physical activity outside school settings among adolescents with overweight and obesity, suggesting that the two settings are relatively independent (Maïano et al., 2018). This might be in line with some of the findings in the qualitative studies, which qualify physical activity in less competitive contexts as safer and more supportive. According to the results of these studies, the school setting seems to be an especially vulnerable context for adolescents with overweight or obesity to be physically active. For some adolescents who are overweight or obese, physical activity at school might be the only social setting for organized physical activity and perhaps an essential setting for forming perceptions of what it is like to take part in physical activity (Sundar et al., 2018).

In terms of gender, the qualitative studies (Chen et al., 2010; Li and Rukavina, 2012; Li et al., 2012; Lewis et al., 2014; Reece et al., 2016; Øen et al., 2018) do not focus on or comment especially on gender. However, among the quantitative studies included, some indicate that girls report more experiences of weight-based victimization than boys and it seems to have a stronger negatively impact on physical activity participation among girls (Puhl and Luedicke, 2012; Stearns et al., 2017). These findings are consistent with others, demonstrating that, among adolescents of all weight categories, teasing and body image concerns may contribute to adolescent girls' reduced rates of participation in sports and other physical activities (Slater and Tiggemann, 2011). However, two of the included quantitative studies did not reveal differences between genders (Watanabe et al., 2017; Maïano et al., 2018). The findings in the quantitative studies are thus inconclusive in term of gender differences and experienced victimization related to physical activity. In average weight girls, teasing experiences in the physical activity context has been linked to lower enjoyment of, and participation in physical activity (Faith et al., 2002). The study by Puhl and Luedicke (2012) also report that girls seem to have stronger reactions toward weight-based victimization than boys. Overall, the number of studies addressing gender differences, among overweight or obese adolescents, regarding experiences of weight-based victimization related to physical activity, is limited and there are questions that needs to be investigated further. An important question is whether the gender differences are particularly pronounced among adolescents who are overweight, as the one included study comprising exclusively adolescents who were overweight or obese did not find differences by sex, or whether it is related to gender in general across weight categories? Furthermore, it will be important to reach a deeper understanding of gender differences in reaction to weight-based victimization and also the causes that lead to possible inequality between the genders.

### Significant Others

Four studies report on adolescents perceived need for social support for being overweight and making changes, as increasing physical activity engagement (Li et al., 2012; Lewis et al., 2014; Reece et al., 2016; Øen et al., 2018). Social support is reported to be sought from family (Lewis et al., 2014), friends (Øen et al., 2018), teachers, and principals (Li et al., 2012). Three studies also indicated that the participants emphasized social connections with other adolescents who were overweight to facilitate engagement in physical activity (Lewis et al., 2014; Reece et al., 2016; Øen et al., 2018). Davison and Schmalz (Davison and Schmalz, 2006) has shown that among adolescents at risk of physical inactivity (including adolescents who are overweight), activity-related support from parents, siblings, and friends may be effective in promoting physical activity participation.

## Implications For Research and Practice

A high number of studies were excluded from this scoping review due to participants being enrolled in treatment at a health-care level not meeting the inclusion criteria for this scoping review. Most of the excluded research focused on and recruited adolescents who were taking part in weight-loss treatments in specialist health service or clinical centers. With only 12 identified studies meeting the inclusion criteria of health-care level in this scoping review, this indicates a need for more research on this topic using community-based samples or samples in the primary health care setting.

Among the included studies, there is little diversity of methods used to examine the links between weight-based victimization and physical activity. All qualitative studies used individual interviews and small focus groups (Chen et al., 2010; Li and Rukavina, 2012; Li et al., 2012; Lewis et al., 2014; Reece et al., 2016; Øen et al., 2018), and all quantitative studies were cross-sectional (Puhl and Luedicke, 2012; Hand et al., 2017; Stearns et al., 2017; Watanabe et al., 2017; Maïano et al., 2018; Himmelstein et al., 2019). Future research should focus on gaining a broader understanding of the phenomenon through research of both aggregative and configurative approach with specific samples of adolescents who are overweight. Future research should also focus on Mixed methods research which can gain a broader understanding of this research phenomenon where the quantitative data can show the strength of associations while the qualitative findings show the nature of those associations (Fetters et al., 2013).

To inform interventions and primary health-care follow-ups that focus on increased physical activity among these adolescents, it might be essential to understand the experiences of weight-based victimization when engaged in physical activity as experienced by adolescents who are overweight or obese, and especially girls. Previous studies have shown that high levels of perceived victimization are significantly related to lower levels of involvement in physical activity (Storch et al., 2006; Gray et al., 2008; Jensen et al., 2013). Being overweight appears to be a factor which predisposes to higher levels of victimization and challenges in forming relations with peers, factors which are important to physical activity engagement (Stankov et al., 2012). Efforts are needed to identify which factors in physical activity may improve engagement in physical activity among these youngsters. That is, a few studies in this scoping review indicated that physical activity in safe and supportive settings with other adolescents with overweight or obesity might improve teenagers' physical activity engagement. More research is needed on this topic to guide future initiatives to engage adolescents who are overweight or obese in physical activity.

Future prevention, identification, and treatment of overweight and obesity in children and adolescents should provide support and empowerment rather than reinforce stigma, shame, or blame. Meland et al. (2021) found that positive self-image and self-esteem protect against weight gain in adolescence (Meland et al., 2021). Therefore, it is important to be aware of the links between weight-based victimization and physical activity for adolescents who are overweight or obese as indicated in this scoping review, and the potential negative consequences these experiences might have on self-image and self-esteem. In addition, for the health-care setting, the concept of physical literacy could help guide interventions aimed at increasing physical activity. School health nurses should consider the holistic factors that could have an impact on physical activity (i.e., enjoyment, motivation, confidence, and knowledge), while identifying the perceived barriers to engagement may facilitate the follow-up of adolescents at risk of inactivity (Cornish et al., 2020).

## Limitations

The links between weight-based victimization and physical activity engagement are not necessarily the main focus of all the studies included in this scoping review, and therefore also other works describing these links in a subtle way might have been excluded due to the search strategy. The inclusions were made because we believe that these studies might contribute to a deeper understanding, and we believe that, through a thorough hand search of bibliographies, we have been able to minimize the risk of selection bias.

Some of the included studies did not differentiate between bullying in general and weight-based bullying, especially in connection with physical activity. Overweight and obese adolescents are at risk of overall bullying, and this might influence physical activity engagement in a different way than those more specific to physical activity.

Due to the nature of scoping reviews, the included studies were not evaluated for quality; thus, in this review, reporting consisted of a direct presentation of the results from the included studies. Several of the studies in this scoping review have small samples, and the results should be interpreted with caution. Still, all qualitative studies in this scoping review point to the same findings, which improves the validity of the papers. Nevertheless, we believe that the included works contribute important perspectives for future research on the association between weight-based victimization and physical activity among adolescents with overweight and obesity.

The inclusion criterion of 13–18 years was set as this is a widely used age range to define adolescents in research. The databases we searched have a filter to set the age of the informants, which we defined as adolescents aged 13–18 years. Nevertheless, many studies on adolescents do not set 13–18 years as an inclusion criterion, which makes it challenging when conducting a review. Some papers might have been excluded from our review, even though some participants might have been 13–18 years old. This might have also led to the inclusion of studies on younger or older participants but in which most participants were in the target age range.

Furthermore, for some studies, it has been challenging to identify the health-care level from where the participants were drawn, for which reason some studies might have been excluded. However, these criteria allowed the authors to narrow the scope of the search to select the most representative studies. Nevertheless, different search criteria might have produced slightly different search results—for example, if dissertations or ongoing research were included. Future research should extend to include more publication types, such as gray literature.

However, the strengths of this scoping review are the systematic nature of the search process and the fact that two authors performed the screening, data extraction, and analysis. The use of a senior librarian to supervise the process of searching for studies was yet another strength of this review.

## Conclusion

A summary of the results in the included studies is somewhat mixed especially in the quantitative paradigm. Still, the majority of the studies in this scoping review has found that there is a high presence of weight-based victimization among adolescents who are overweight or obese and this seems to have a negative impact on engagement in physical activity. The included qualitative studies gave us an opportunity to elaborate especially on the adolescents' experiences which revealed a high presence of teasing, bullying, insecurity of appearance, inferiority, and negative assumptions from peers when engaged in physical activity. This seems to negatively affect the adolescent's engagement in physical activity, probably as a coping reaction to avoid weight-based victimization.

These results indicate that research should focus on how to facilitate engagement in physical activity in several contexts for adolescents who are overweight or obese to be able to successfully decrease inactivity. Increased awareness of weight-based victimization and its consequences is needed in the prevention, identification, and treatment of overweight and obesity among children and adolescents.

This scoping review reveals important gaps, which are vital to address in future research in the field. In general, there is a need for a broader knowledge of links between weight-based victimization and physical activity. Currently, research in the field is characterized by homogeneous research methods; cross sectional design (quantitative) and interview studies (qualitative). To achieve deeper understanding, we suggest there is a need for studies that combine quantitative and qualitative methods with a mixed method approach. In addition, we find a need for research with specific samples, where the research focus is specifically on the group of overweight and obese adolescents. In relation to gender, the findings indicate that girls are more vulnerable than boys, but there are few studies, and the findings are mixed. We therefore consider it important to investigate gender differences more closely. Summarized, this scoping review demonstrate a need for more diverse research with specific aims and scopes to understand the associations between weight-based victimization and physical activity.

## Author Contributions

IBS led the development of the manuscript. IBS and KLH collaborated in developing the inclusion/exclusion criteria, conducted article screening, data analysis, and drafting of the manuscript. FOB and RJK edited continuous iterations of the manuscript draft and provided input on the direction of the data discussion. All authors contributed to the article and approved the submitted version.

## Funding

The article is part of a Ph.D. study funded by Volda University College, by paying the Ph.D. student's salary. No additional funding.

## Conflict of Interest

The authors declare that the research was conducted in the absence of any commercial or financial relationships that could be construed as a potential conflict of interest.

## Publisher's Note

All claims expressed in this article are solely those of the authors and do not necessarily represent those of their affiliated organizations, or those of the publisher, the editors and the reviewers. Any product that may be evaluated in this article, or claim that may be made by its manufacturer, is not guaranteed or endorsed by the publisher.
