# Supplementary material for: Weight-Based Victimization and Physical Activity Among Adolescents With Overweight or Obesity: A Scoping Review of Quantitative and Qualitative Evidence
Source: Front Sports Act Living. 2022 Jan 28;4:732737. doi: 10.3389/fspor.2022.732737 (PMC8832147; doi:10.3389/fspor.2022.732737)

# Additional file 1. Search strategy

PubMed Search Strategy (Literature search performed: November 20, 2020)


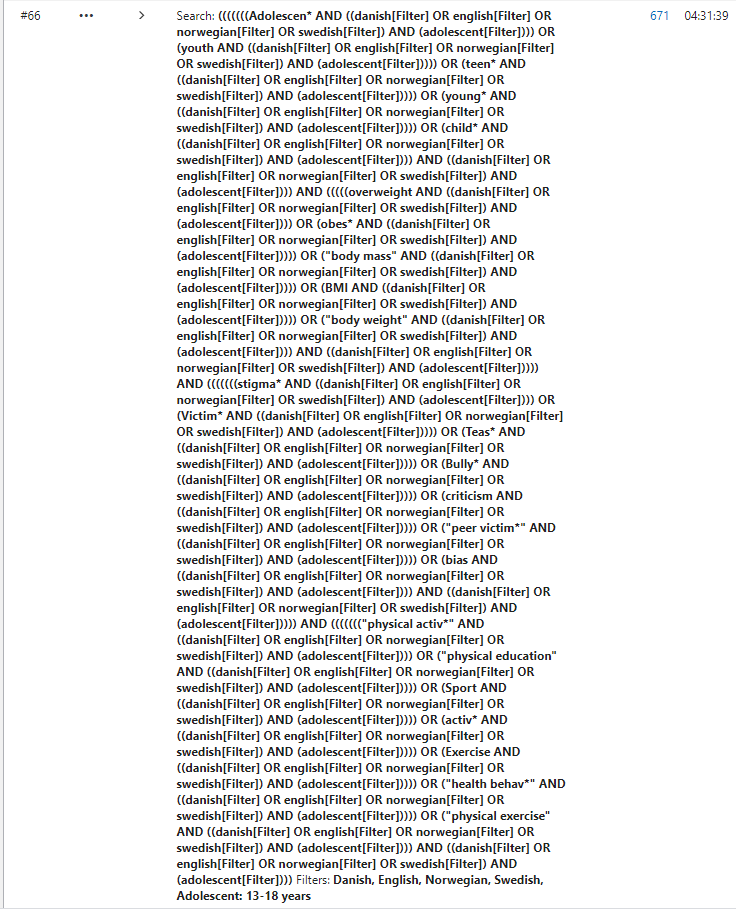

Supplement: Supplementary file 2 [file Table_1.DOCX]
